# Development and Characterization of an In Vitro Round Window Membrane Model for Drug Permeability Evaluations

**DOI:** 10.3390/ph15091105

**Published:** 2022-09-05

**Authors:** Ruby Singh, Bhaskar Birru, Joachim G. S. Veit, Elizabeth M. Arrigali, Monica A. Serban

**Affiliations:** 1 Department of Biomedical and Pharmaceutical Sciences, University of Montana, 32 Campus Dr., Skaggs 394, Missoula, MT 59812, USA; 2 Montana Biotechnology Center (BIOTECH), University of Montana, Missoula, MT 59812, USA

**Keywords:** round window membrane, airway cells, drug permeation, in vitro models

## Abstract

Hearing loss and balance disorders are highly common disorders, and the development of effective oto-therapeutics remains an area of intense research. Drug development and screening in the hearing research field heavily rely on the use of preclinical models with often ambiguous translational relevance. This often leads to failed advancement in the market of effective therapeutics. In this context, especially for inner ear-specific pathologies, the availability of an in vitro, physiologically relevant, round window membrane (RWM) model could enable rapid, high-throughput screening of potential topical drugs for inner ear and cochlear dysfunctions and could help accelerate the advancement to clinic and market of more viable drug candidates. In this study, we report the development and evaluation of an in vitro model that mimics the native RWM tissue morphology and microenvironment as shown via immunostaining and histological analyses. The developed three-dimensional (3D) in vitro model was additionally assessed for barrier integrity by transepithelial electrical resistance, and the permeability of lipophilic and hydrophilic drugs was determined. Our collective findings suggest that this in vitro model could serve as a tool for rapid development and screening of topically deliverable oto-therapeutics.

## 1. Introduction

Hearing loss and balance disorders affect 466 million individuals worldwide, with the figure estimated to rise to 900 million by 2050 [1]. Various factors such as chronic infections, noise exposure, chronic diseases, the aging process, genetic mutations, and ototoxic drugs affect hearing loss and balance disorders. Hearing loss is predominantly an irreversible process, and there are currently no approved medications or treatments available that target inner ear sensory dysfunction or sensory recovery [2,3]. Cochlear implants and hearing aids are intended for the management of hearing loss but are not a complete cure. Therefore, the development of novel drug candidates and localized drug delivery methods for the treatment of hearing loss is an essential unmet need in the field. Nevertheless, localized drug delivery to the inner ear and cochlear structures affected by hearing loss is challenging, which severely hampers the advancement of effective drugs in clinical settings [3,4,5].

The round window membrane (RWM) is a non-osseous barrier that separates the middle and inner ear compartments, potentially making it an ideal portal for topically delivering medications to the inner ear [6,7]. As a result, understanding and anticipating RWM permeation efficiency could provide useful information for improving local drug delivery with clinical significance [8,9,10]. Multiple studies have been conducted to assess the RWM permeability of various drugs in animal models, some supplemented with computational modeling. However, these preclinical models result in long drug development times, high costs, and in certain instances, have limited clinical translational relevance [11,12,13,14].

**Figure 1:**
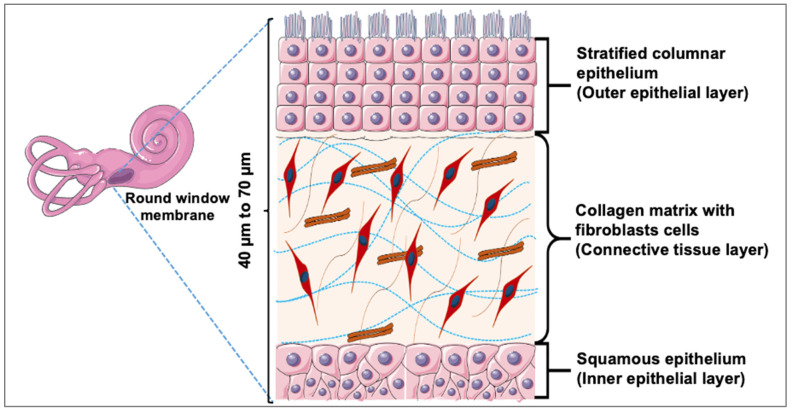
**Illustration of Round Window Membrane Cross Section**. The outer layer of the RWM consists of stratified columnar epithelial cells. The middle layer consists of fibroblasts embedded in connective tissue (primarily collagen and elastin). The inner layer of the RWM is made up of thin squamous epithelial cells. This figure was drawn using modified artwork from Servier Medical Art. Servier Medical Art by Servier is licensed under a Creative Commons Attribution 3.0 Unported License (https://creativecommons.org/licenses/by/3.0/) (accessed on 10 July 2021).

With the limitations of the existing preclinical models, numerous research areas have recently employed the use of three-dimensional (3D) in vitro models to accelerate and improve the drug discovery process [15,16,17,18,19,20,21,22]. This approach has been recognized by the Food and Drug Administration as a viable tool for drug discovery advancement [16]. Similarly, the availability of physiologically relevant 3D models of the human inner ear structures could accelerate the discovery of therapeutic agents specifically targeting the structures affected by hearing loss. A viable model of the RWM could allow for rapid assessment of topically deliverable drugs or drug delivery systems. A simplified mouse tracheal cell layer model employed in one drug permeation study showed encouraging results and underlined the feasibility of this approach [23]. For the development of physiologically relevant in vitro RWM drug permeation models, a key step is understanding the membrane’s architecture and its impact on drug permeation. Histologically, the RWM consists of an outer epithelial layer, which interfaces with the air-filled middle ear; a central or middle layer, which is made up of elastic fibers and collagen-rich connective tissue; and an inner epithelial layer, which interacts with scala tympani—a fluid-filled cochlear compartment (Figure 1) [24,25].

In the present study, we report the development and evaluation of such an engineered in vitro 3D RWM model for drug permeability evaluation studies. Individual RWM components were histologically characterized, their barrier properties were assessed, and their contributions to drug permeability were evaluated. A panel of four drugs with a range of lipophilic to hydrophilic partition coefficients (log P) was evaluated (Supplemental Table S1). Our findings indicate that RWM drug diffusion rates are primarily dictated by the outer layer of stratified columnar epithelial cells, which we modeled with primary human small airway epithelial cells. These data seem in agreement with previous reports indicating that the outer epithelium is the primary contributor to the physiological RWM barrier properties [26] and highlight the feasibility of using these engineered in vitro models as rapid, high-throughput screening tools for oto-therapeutic development.

## 2. Results

### 2.1. Modeling of the Outer Layer of the RWM

**Figure 2:**
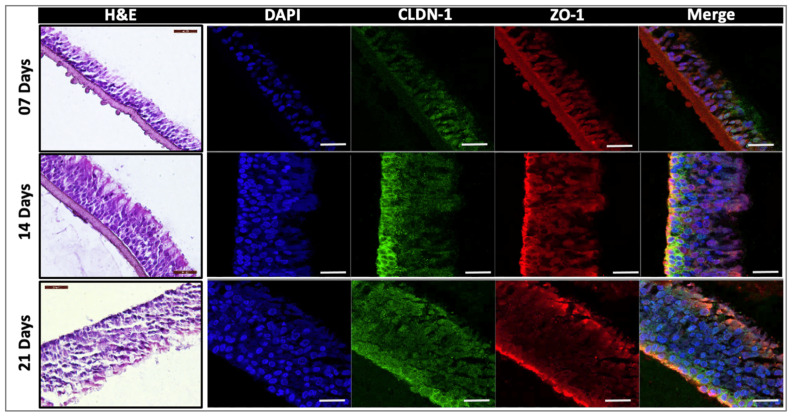
**Histological and immunofluorescent evaluation of 3D airway constructs**. H&E (left), nuclear (DAPI, blue), CLDN-1 (green), and ZO-1 (red) staining of airway tissues grown while exposed to air–liquid interface for 7, 14, or 21 days (*n* = 4 for each time point). Scale bar 50 μm for H&E; scale bar 20 μm for immunofluorescence. ALI, air–liquid interface; CLDN-1, claudin-1; DAPI, 4′,6-diamidino-2-phenylindole; H&E, hematoxylin and eosin; ZO-1, zona occludens-1.

Primary human airway epithelial cells were cultured for up to 21 days at air–liquid interface (ALI), with the basal side of the cells in contact with culture media and the apical side exposed to air. To assess the quality of the generated airway epithelia tissues (AETs), histological evaluations were performed on the airway epithelial tissue model at three distinct culture time points (7, 14, and 21 days) after ALI exposure (Figure 2).

**Figure 3:**
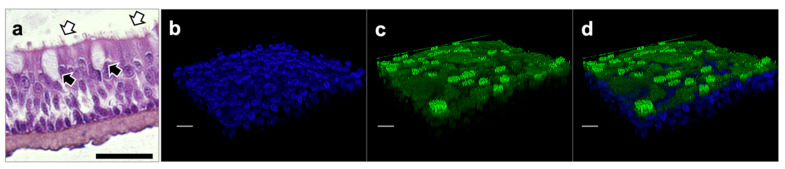
**Assessment of tissue ciliation.** (**a**) H&E staining with white arrows indicating cilia and black arrows indicating goblet cells; (**b**) nuclear (DAPI, blue); (**c**) occludin (green); and (**d**) merged stained tissues with top confocal microscope view of hair cells. Tissues at day 14 ALI culture. Scale bar 50 μm for H&E; scale bar 20 μm for immunofluorescence. ALI, air–liquid interface; DAPI, 4′,6-diamidino-2-phenylindole; H&E, hematoxylin and eosin.

The analysis of the constructs via hematoxylin and eosin (H&E) staining indicates that the thickness of the tissue gradually increased throughout the culture period. In addition to the morphological evaluations, the presence of tight junctions, essential for the evaluation of the permeability barrier functions of the formed tissues, was evaluated. Two tight junction markers were used for this: claudin-1 (CLDN-1, green) and zona occludens-1 (ZO-1, red). ZO-1 was predominantly observed close to the apical surface of the cells joining the columnar cells together when cells were viewed in cross-section (Figure 2). Both CLDN-1 and ZO-1 showed poor and discontinuous expression on day 7 in ALI cultures, indicative of immature tissues; however, their expression was high on days 14 and 21, respectively (Figure 2), consistent with expected tissue maturation. High-resolution images of H&E-stained constructs of day 14 in ALI cultures also revealed the presence of both ciliated and goblet cells (Figure 3a), consistent with expected 3D airway tissue development and maturation. The ciliation of the cultured cells was also confirmed via confocal microscopy (Figure 3b–d). Overall, the histological and immunofluorescent evaluation of the airway constructs showed adequate tissue maturation, with cellular ciliation and differentiation, as well as tight junction formation.

**Figure 4:**
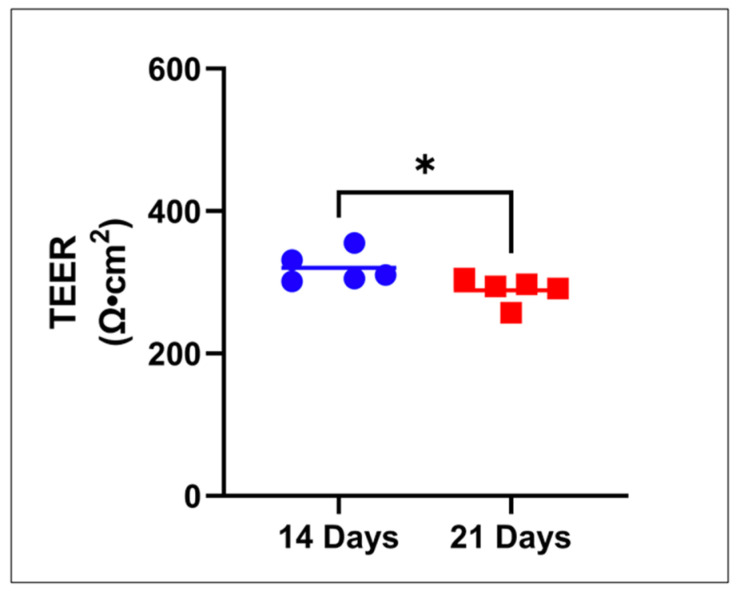
**Tissue barrier properties of AET constructs.** Constructs at day 14 and 21, respectively, in culture, as evaluated by transepithelial electrical resistance (TEER) measurements. Unpaired *T* test, *p < 0.05, *n* = 5. AET, airway epithelial tissues.

The drug permeability properties of the engineered RWM layers were assessed next. Transmembrane epithelial electrical resistance (TEER) measurements were performed as a first evaluation of the AET construct tissue barrier properties (Figure 4).

**Figure 5:**
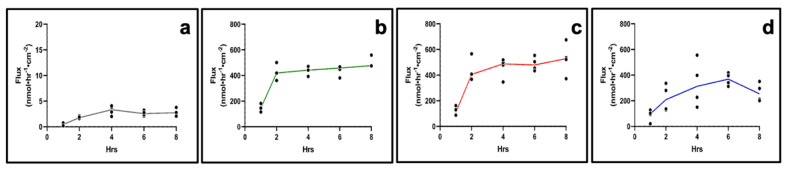
**Drug permeation in day 14 AET constructs**. (**a**) Fluorescein, (**b**) DSP, (**c**) ciprofloxacin HCl, (**d**) gentamicin sulfate. *n* = 4 for all drugs; lines show mean. AET, airway epithelial tissues; DSP, dexamethasone sodium phosphate.

An average TEER of 331 ± 14 Ω.cm^2^ and 305 ± 11 Ω.cm^2^ was recorded for day 14 and day 21, respectively, of AET culture, with a statistical significance between values. Next, the permeability of four drugs with different lipophilic/hydrophilic properties, grown for 14 or 21 days, was assessed in AET. On day 14, steady state (SS) was reached after 2 h in all drugs (Figure 5).

**Figure 6:**
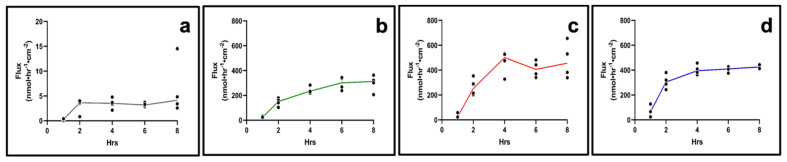
**Drug permeation in day 21 AET constructs**. (**a**) Fluorescein, (**b**) DSP, (**c**) ciprofloxacin HCl, (**d**) gentamicin sulfate. *n* = 4 for all drugs; lines show mean. AET, airway epithelial tissues; DSP, dexamethasone sodium phosphate.

Flux was the lowest for fluorescein, the most lipophilic drug (log P = 3.88; flux_ss_ = 2.38 nmol·hr^−1^·cm^−2^, P_app_ = 2.89 × 10^−2^ cm/hr). Dexamethasone sodium phosphate (DSP, log P = 1.56) and ciprofloxacin HCl (log P = 0.86) had comparable flux values (flux_ss_ = 442.44 nmol·hr^−1^·cm^−2^, P_app_ = 8.68 × 10^−2^ cm/hr for DSP and flux_ss_ = 460.65 nmol·hr^−1^·cm^−2^, P_app_ = 7.63 × 10^−2^ cm/hr for ciprofloxacin HCl), while for gentamicin sulfate (log P = −3.1) the SS appears to have been reached after 2 h (flux_ss_ = 349.37 nmol·hr^−1^·cm^−2^, P_app_ = 5.56 × 10^−2^ cm/hr). At day 21, the SS was reached after 4 h for all drugs (flux_ss_ = 3.36 nmol·hr^−1^·cm^−2^, P_app_ = 3.36 × 10^−2^ cm/hr for fluorescein; flux_ss_ = 222.36 nmol·hr^−1^·cm^−2^, P_app_ = 4.36 × 10^−2^ cm/hr for DSP; flux_ss_ = 450.39 nmol·hr^−1^·cm^−2^, P_app_ = 7.46 × 10^−2^ cm/hr for ciprofloxacin HCl), and gentamicin sulfate, (flux_ss_ = 403.93 nmol·hr^−1^·cm^−2^, P_app_ = 6.43 × 10^−2^ cm/hr) (Figure 6). The analysis of the apparent permeability coefficients (P_app_) for the four drugs on day 14 and day 21 tissues indicated that the day 14 constructs are more selective towards the tested drugs compared to day 21 constructs (Supplemental Figure S1).

### 2.2. Modeling the Middle Layer of the RWM

**Figure 7:**
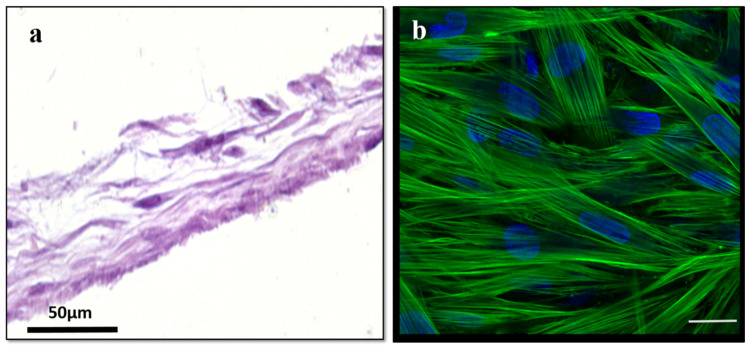
**Histological and immunofluorescent evaluation of 3D PFCs**. (**a**). H&E (left), (**b**) nuclear (DAPI, blue), and actin (green) staining of day 28 fibroblast-based connective tissues. Scale bar 50 μm for H&E; scale bar 20 μm for immunofluorescence. DAPI, 4′,6-diamidino-2-phenylindole; H&E, hematoxylin and eosin; PFCs, primary fibroblast constructs.

**Figure 8:**
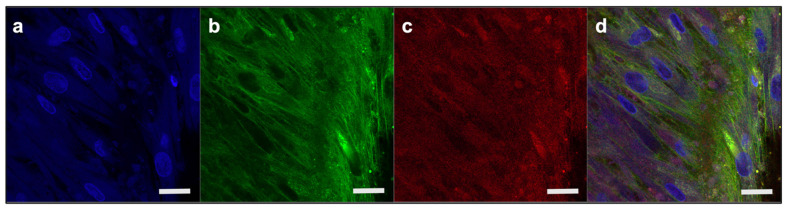
**Extracellular matrix deposition in PFCs**. (**a**) nuclear (DAPI, blue); (**b**) fibronectin (green); and (**c**) collagen-1 (red); (**d**) merged image. Scale bar 20 µm. DAPI, 4′,6-diamidino-2-phenylindole; PFCs, primary fibroblast constructs.

The middle connective tissue layer of the RWM was modeled by culturing primary fibroblasts for 28 days. The quality of the formed tissues was assessed via histology and immunostaining (Figure 7). The maturity of the primary fibroblast constructs (PFCs) was also evaluated via extracellular matrix deposition, specifically for the presence of collagen-I and fibronectin (Figure 8). Collectively, the data indicate the formation of established connective tissues similar to the native RWM middle layer.

**Figure 9:**
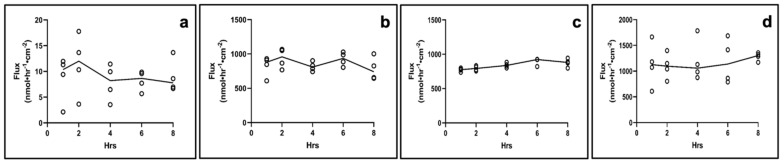
**Drug permeation in day 28 PFCs**. (**a**) Fluorescein, (**b**) DSP, (**c**) ciprofloxacin HCl, (**d**) gentamicin sulfate. *n* = 4 for all drugs; lines show mean. DSP, dexamethasone sodium phosphate; PFC, primary fibroblast culture.

The drug permeation properties in PFCs were also evaluated. Compared to the permeability values observed in AETs on days 14 and 21, respectively, the PFCs showed higher flux values for all the drugs but maintained the same selectivity towards the drugs tested (Figure 9). These observations are also aligned with the recorded PFC TEER values, which were negligible compared to control/no tissue values, indicating that the connective tissue layer did not have a substantial barrier effect.

### 2.3. Effect of Freeze/Thaw Cycles on Barrier Properties of the In Vitro RWM Outer Layer Model

**Figure 10:**
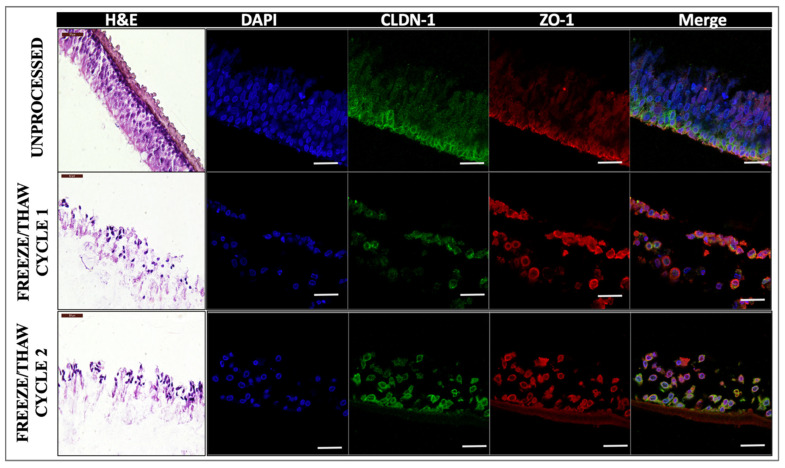
**Histological and immunofluorescent evaluation of AET constructs subjected to freeze/thaw cycles**. H&E (left), nuclear (DAPI, blue), claudin-1 (CLDN-1, green), and zona occludens-1 (ZO-1, red) staining of unprocessed (top row), freeze/thawed once (middle row), or freeze/thawed twice (bottom row). Scale bar 50 μm for H&E; scale bar 20 μm for immunofluorescence; *n* = 4. AET, airway epithelial tissue; CLDN-1, claudin-1; DAPI, 4′,6-diamidino-2-phenylindole; H&E, hematoxylin and eosin; ZO-1, zona occludens-1.

Tissue integrity is typically compromised by freeze/thaw processes. Therefore, we sought to understand the effect of one versus two freeze/thaw cycles on the quality of the tissue, its barrier effects, and drug permeability. Histological and immunohistological analyses of tissues subjected to one or two freeze/thaw cycles were performed. Respectively, freeze/thaw cycles showed a compromised barrier, with discontinuous apical and epithelial layers and more porous structure, as well as irregular tight junctions compared to the fresh samples (Figure 10). The typical thick and continuous layer of CLDN-1 on the apical layer of unprocessed tissues was affected in the frozen samples and was more irregular in twice frozen/thawed tissues.

**Figure 11:**
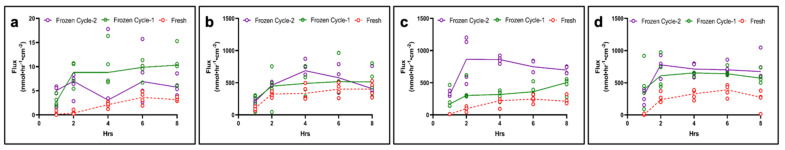
**Drug permeation in day 14 AET constructs subjected to freeze/thaw cycles**. (**a**) Fluorescein, (**b**) DSP, (**c**) ciprofloxacin HCl, (**d**) gentamicin sulfate. *n* = 4 for all drugs; lines show mean. AET, airway epithelial tissue; DSP, dexamethasone sodium phosphate.

In terms of drug permeability, freeze/thawing increased the flux across AETs for all four drugs compared to fresh samples (Figure 11). This observation was consistent with a significant drop in AET TEER values in frozen tissues (Supplemental Figure S2).

## 3. Discussion

The present study sought to develop in vitro 3D RWM models to enable the rapid, high-throughput screening of novel drug candidates against inner ear pathologies. Our approach was to individually engineer and evaluate tissues corresponding to the constituent layers of the native RWM. For the outer layer, we employed human primary airway epithelial cells to form 3D constructs that would mirror the histology of the native tissue layer. For the middle connective tissue layer, we used primary human dermal fibroblasts and allowed them to mature into tissues via endogenous extracellular matrix secretion. For the inner layer, we attempted to model it using human primary renal proximal tubule epithelial cells, which have previously been studied for this purpose [27], as potential representatives of the native tissue layer; however, no confluent monolayers were obtained, and the relevance of using these cells to model RWM components is unclear. Additionally, it has been suggested that this inner layer is not expected to play a significant role in RWM permeability relative to the outer epithelial layer [28].

With the 3D outer and middle layers modeled as AET and PFC, we next assessed the drug permeation properties of four different drugs with a wide range of permeability coefficients. Three of the drugs (DSP, ciprofloxacin HCl, and gentamicin sulfate) are currently used or have been historically used for the treatment of otic pathologies, while fluorescein was chosen because of its pronounced lipophilicity and ease of detection. The permeability data indicate that the RWM models allow selective drug permeation, which we postulate is largely dictated by log P and molecular weight [29]. Highly lipophilic molecules such as fluorescein molecular weight (MW = 332.3 g/mol, log P = 3.88) [30] or hydrophilic drugs such as gentamycin sulfate (MW = 1488.8 g/mol, log P = −3.1) [31] showed decreased transmembrane permeation rates compared to the more lipophilic/hydrophilic balanced drugs such as DSP (MW = 516.4 g/mol, log P = 1.56) [32] or ciprofloxacin HCl (MW = 367.8 g/mol, log P = −0.86) [33], that more readily permeated the tissues.

Histologically, the outer layer of the human RWM consists of a permeable [8], single cell layer outer epithelium, a middle connective tissue layer, and an inner layer of squamous cells towards the inner ear [34]. Previous drug permeability reports suggest that in RWM, the outer epithelial layer is the key determinant of the membrane’s permeation properties [26]. This finding seems to be reflected by our in vitro 3D models where the permeation through the AETs, representative of the native RWM outer layer, is the rate-limiting step, compared to the permeation through the PFCs, which represent the connective tissue, middle layer of the native RWM. Although we did not directly test a model of the inner layer, we are confident that the impact of such a construct on various drug permeation rates would be insignificant, as the human inner layer of the RWM was reported to be discontinuous and with loose junctions [26]. Therefore, our data indicate that an outer layer-mimicking 3D model would be sufficient in vitro representation of the RWM in terms of drug permeability.

For AETs, two different culture times were evaluated. The barrier properties (TEER values) of the tissues at the two culture points were statistically significant. Day 14 tissues appeared to have a higher selectivity towards drugs based on their log P and MW; however, compared to other tissue constructs are known for their barrier properties (i.e., skin or the blood-brain-barrier) [35,36] the observed TEER differences between the two culture times do not seem pharmacokinetically relevant. The day 21 AETs seemed less selective for drugs, although they still showed the lowest permeation rate for the drug with the highest lipophilicity (fluorescein). The final selection of the most representative in vitro model will have to rely on future studies of human RWM drug permeation.

We also assessed the impact of freeze/thaw cycles on the tissue integrity and drug permeation properties of our in vitro 3D models as in situ tissue drug permeation studies showed that in the tympanic membrane (TM), the barrier properties of tissues are affected by such processing parameters. Our results indicated that freeze/thawing does indeed affect the quality of the tissue [37]; however, the drug diffusion properties are not significantly altered, most likely due to the permeable nature of the outer layer of RWM compared to the multicellular outer layer structures of the TM [8,29].

Overall, we showed that engineering an outer layer 3D model is sufficient for the assessment of the permeability properties of different drugs, as observed in native tissues [26,38]. These models histologically mimic the outer layer of the RWM. Moreover, the models, especially the day 14 constructs, seem to discern between different drugs based on their partition coefficient and molecular weight, in agreement with previously published reports [29]. Considering the robustness of the 3D AET constructs, we believe that these tissues could represent a reliable tool for the rapid, high-throughput screening of novel topically deliverable drug candidates targeting various inner ear pathologies. If validated with human RWM studies, such in vitro models have the capability of accelerating the discovery of novel, effective, topically deliverable therapeutics and could minimize the need for animal models in the early stages of drug development and discovery.

## 4. Materials and Methods

### 4.1. Tissue Cultures

Primary human small airway epithelial cells (HSAEC; normal (ATCC PCS301010, Manassas, VA, USA) were cultured in airway epithelial cell basal media supplemented with bronchial epithelial cell growth kit (ATCC PCS300040, Manassas, VA, USA) components. PneumaCult™-ALI complete base medium (PALI, Manassas, VA, USA) (PneumaCult™-ALI 10X Supplement + PneumaCult™-ALI Basal Medium supplemented with PneumaCult™-ALI Maintenance Supplement Heparin Solution and Hydrocortisone Stock Solution) was used for air–liquid interface (ALI). Cells were grown in T-75 flasks up to 80% confluence in an incubator at 37 °C, with 5% CO_2._ The culture medium was changed every two days. Once cells were 70–80% confluent, they were released, transferred to a conical tube, and pelleted via centrifugation at room temperature (RT) for 5 min at 150 RCF. The supernatant was aspirated, and cells were resuspended in fresh PALI. PALI was added to the 12-well plates (basal chamber), and cell suspension (10^5^ cells/insert) was placed on the cell culture insert (apical chamber). Media change was performed after 24 h in both basal and apical chambers, and 72 h after plating, media change was conducted, and media was added only to the basal chamber. Full media change was performed in the basal chamber using PALI every 2 days, leaving the apical chamber empty. The tissues were allowed to grow for different time points (7, 14, and 21 days after establishing ALI), and at the end of each time point, experiments were performed. Excess mucus was removed from the apical surface by washing the cells with DPBS at RT.

Primary human dermal fibroblasts (HDF, human, primary, ATCC PCS-201-012, Manassas, VA, USA) were grown for 28 days to form a uniform layer of HDF cells. Morphology of the HDF cells was analyzed by staining the cells for actin filaments [39,40].

### 4.2. Histology, Immuno-Labeling, and Imaging of Tissues

Fixing solution (4% formaldehyde + 1% acetic acid in PBS) was used to fix the tissues overnight and then processed and paraffin-embedded using a Leica ASP300S tissue processor. Sections of 6 µm were taken on glass slides and were either stained with hematoxylin and eosin (H&E) or deparaffinized and rehydrated for immuno-labeling in a Leica Autostainer XL.

For immunostaining, deparaffinized tissues were washed with PBS and permeabilized with 0.5% Triton X-100 in PBS for 20 min, washed 3 × in PBS, and blocked with 3% BSA in PBS for 30 min at RT. After blocking cells were washed with PBS thrice and were stained with primary antibodies overnight at 4C, washed 3 × in PBS, and then incubated with secondary antibodies prepared in 3% BSA in PBS for 1 h at RT. After secondary antibody staining, cells were washed with PBS twice and counterstained with DAPI for 15 min and washed thrice with DPBS. After washing cells were mounted using Mowiol + DABCO mounting media (10% *w*/*v* Mowiol 4-88, 25% *w*/*v* glycerol, 0.1 M Tris (pH 8.5), and 2.5% *w*/*v* 1,4-diazobicyclo-[2.2.2]-octane) on coverslips. Tissue was observed under the confocal microscope Leica Stellaris 5 Confocal Microscope (Leica, Germany).

Primary antibodies goat anti-human ZO-1 (Invitrogen, PA5-19090, Waltham, MA, USA), rabbit anti-human CLDN-1 (Invitrogen, 51-9000, Waltham, MA, USA), and rabbit anti-human Occludin (Invitrogen, 40-4700, Waltham, MA, USA) were diluted 1:200. Secondary antibody donkey anti-goat 647 (Invitrogen, A32849, Waltham, MA, USA) was diluted 1:500 and donkey anti-rabbit 555 (Invitrogen, A32794, Waltham, MA, USA) was diluted 1:1000. DAPI (Thermo Scientific, 62248, Waltham, MA, USA) was diluted 1:1000.

To evaluate the morphology of human dermal fibroblast tissue, it was stained with actin dye (ActinRed 555, Invitrogen, R371121 drop/mL, Waltham, MA, USA) for actin filaments and counter stained with DAPI for nuclei. Extracellular matrix deposition was assessed for collagen I (Anti-Collagen Type I antibody, Rockland, 600-401-103S Pottstown, PA, USA) and fibronectin (Fibronectin Polyclonal Antibody, Invitrogen, PA5-29578, Waltham, MA, USA).

### 4.3. Permeation Devices

An in-house permeation device was employed for drug permeation studies, and transepithelial electrical resistance (TEER) measurements were used to identify potential leakage between the interface of the cell culture insert and the tissue layers. The permeation devices were designed in Fusion360 software (Autodesk, San Rafael, CA, USA), exported to PreForm software, and then printed using Clear V4 resin on a Form 3 photopolymer 3D printer (Formlabs, Somerville, MA, USA). The permeation device was made to fit within a standard 6-well cell culture plate serving as the receiver chamber and holding a standard 24-well plate cell culture insert. The donor chamber (6 mm inner diameter) is placed over the tissue-containing insert, which is placed into the base unit of the permeation device. The donor and receiver chambers are sealed off by the top unit, which has a 6 mm inner diameter “donor” chamber, which is put over the tissue and fastened in place with three stainless steel M2 bolts screwed into base unit nuts that had been press-fit into the base unit. While the second electrode is being placed into the donor chamber, a secondary channel going to the receiver chamber allows for the placement of a TEER electrode.

### 4.4. TEER Measurements

TEER is widely accepted as an index of the integrity of tight junctions. TEER was measured for the airway tissue prior to permeation testing with a Millicell ERS-2-volt ohmmeter (Millipore, MERS00002, Burlington, MA, USA). The tissue culture insert was placed gently with the help of tweezers into the well. To measure the TEER in the insert, 1500 µL of DPBS was placed into the well of a 12-well plate. Then, 500 µL of DPBS was added into the insert (over top of tissue), and the electrode was placed (MERSSTX01). The longer electrode was placed outside of the insert; the shorter electrode was placed into the insert over the tissue, and the electrode was not touching the base of the well. The values were normalized with the surface area and expressed as Ω.cm^2^.

### 4.5. Drug Preparation

Fluorescein (Fluka Analytical, 32615-25G-R, St. Louis, MO, USA) was prepared at 10 mM concentration (equivalent to 3.323 mg/mL) and diluted to 100 µM. pH was adjusted to 8.5 ± 0.5 with 1 M hydrochloric acid. Dexamethasone 21-phosphate disodium (Alfa Aesar, J64083, Tewksbury, MA, USA) was prepared at 2.63 mg/mL (equivalent to 0.2% *w*/*v* dexamethasone) in phosphate-buffered saline (PBS). Ciprofloxacin hydrochloride monohydrate (Alfa Aesar, J61970, Tewksbury, MA, USA) was prepared at 2.33 mg/mL (equivalent to 0.2% *w*/*v* ciprofloxacin) in PBS, and the pH was adjusted to 3.5 ± 0.5 with 1 M hydrochloric acid. Gentamicin Sulfate was prepared at 3.62 mg/mL equivalent to 3 mg/mL) in PBS. The given concentration for all four drugs was chosen on the basis of clinically available formulations except fluorescein.

### 4.6. Drug Permeation Testing

After TEER measurement, airway tissue was placed into a 6-well plate containing 2500 µL PBS (receiver solution), and 200 µL of drug solution was placed into the donor chamber, being sure that no air bubbles became trapped above or below the tissue.

At each time point (1, 2, 4, 6, 8, and 24 h), the device was removed from the well, allowed to drip, then transferred to a new well containing fresh donor solution. To ensure a consistent gradient, 100 µL of drug solution was removed from the donor channel and replaced with the fresh drug at each time point. All receiver solutions were collected immediately in microfuge tubes and stored at −20 °C until analysis. Any noticeable decrease in donor solution level in tissue indicated a leak or compromised tissue, and the sample was excluded from the study.

Fluorescein was used as a model drug for drug permeability testing, and it can be traced easily due to its fluorescent property. The permeability of Fluorescein was quantified using Citation. Concentrations of Fluorescein were determined by measuring the fluorescence intensity of the apical samples using a Cytation 5 (Biotek, Winooski, VT, USA) microplate reader (ex. 490 nm; em. 515 nm) against a standard curve. Other drugs were quantified using the HPLC method.

### 4.7. High-Performance Liquid Chromatography

The flux measurement and drug permeability in the time-dependent permeation were determined using HPLC. All samples were filtered with 0.22 µm PVDF syringe filters prior to HPLC analysis. All reagents used were HPLC-grade; the buffers were freshly made and filtered at 0.1 µm prior to use. HPLC was performed using an Agilent 1260 Infinity II system (Agilent Technologies Inc., Santa Clara, CA, USA).

Dexamethasone sodium Phosphate: 50 µL of the sample was injected into an isocratic mobile phase of 75% 0.01 M potassium phosphate buffer (pH 7.58) and 25% acetonitrile flowing at 1.5 mL/min through a Gemini 3 µm 100 × 4.6 mm reverse-phase C18 column (Phenomenex, 00D-4439-E0) protected by a Security Guard C18 4 × 3.0 mm cartridge (Phenomenex, AJ0-7597) at 25 °C. The retention time of DSP at 1.9 min was used to quantify the area under the curve (AUC) of absorbance at 240 nm.

Ciprofloxacin: 50 µL of the sample was injected into an isocratic mobile phase of 80% 0.02 M potassium phosphate buffer (pH to 2.7 with orthophosphoric acid) and 20% acetonitrile flowing at 1.0 nmL/min through a Gemini 5 µm 150 × 3 mm reverse-phase C18 column (Phenomenex, 00F-4435-Y0, Torrance, CA, USA) protected by a Security Guard C18 4 × 2.0 mm cartridge (Phenomenex, AJ0-7596, Torrance, CA, USA) at 25 °C. The retention time of ciprofloxacin at 1.75 min was used to quantify the area under the curve (AUC) of absorbance at 277 nm.

Gentamicin Sulfate: Gentamicin was pre-column derivatized using the o-phthalaldehyde reaction. The filtered sample of 50 μL is combined with 55 μL of isopropanol and 20 μL of Reagent B (1 mL HPLC-grade methanol, 19 mL Reagent A, 0.4 mL thioglycolic acid, 200 mg O-phthalaldehyde, pH adjusted to 10.4 using 8 N potassium hydroxide), vortexed for 10 s and then incubated at 60 °C for 15 min. The reagent A is 0.4 M boric acid adjusted to a pH of 10.4 with 8N potassium hydroxide. Following the derivatization procedure, the samples were subjected to HPLC analysis. Then, 50 µL of the sample was injected into an isocratic mobile phase of 100% (5 g/L sodium 1-heptane sulfonate monohydrate in 70:25:5 methanol–water–acetic acid) flowing at 0.8 mL/min through a Gemini 5 µm 150 × 3 mm reverse-phase C18 column (Phenomenex, 00F-4435-Y0, Torrance, CA, USA) protected by a Security Guard C18 4 × 2. 0 mm cartridge (Phenomenex, AJ0-7596) at 25 °C. The retention time of C-components of gentamicin at C1a − 7.9 min; C2a − 10.3 min; C2 − 11.9 min were used to quantify the area under the curve (AUC) of absorbance at 330 nm.

### 4.8. Analysis of Flux and Apparent Permeability Coefficient

Following the amount of drug permeated through tissues was determined, the flux was calculated using Flux=D ÷ΔT÷A, where D is the drug permeated since the previous time point (nmoles), A is the permeable area (cm^2^), and ΔT is the elapsed time (hours) since the previous time point.

Using Fick’s law, the apparent permeability coefficient (P_app_, cmhr-1) was calculated:

$$P_{\mathrm{app}}=\frac{{\mathrm{Flux}}_{\mathrm{ss}}}{C_{d}-C_{r}}$$

where C_r_ is the receiver solution concentration (considered constant at 0 since receiver solution was replaced at each time point and results confirm receiver solution remained negligible), and Flux_ss_ is the average of the flux values once a steady state has been reached. C_d_ is the donor solution concentration (nmol.cm^−3^), which is assumed to be constant due to excess and regular replenishment of donor solution.

### 4.9. Tissue Freeze/Thawing Process

For freeze/thawing processing, samples (*n* = 4) were frozen for 24 h at −20 °C (cycle 1) and for cycle 2, frozen for 24 h, after 24 h allowed to thaw for 30 min, then refrozen for an additional 24 h at −20 °C. Post-freezing, samples were taken out and allowed to thaw completely. Subsequently, tissue integrity and drug permeability analyses were performed as described above. Unprocessed samples (not frozen/thawed) were used as controls.

### 4.10. Statistical Analyses

All experiments were run in triplicates or greater, and all data were presented as mean ± standard deviation of the mean. Student *t*-tests were performed for 2-group comparisons, while one-way ANOVA with multiple comparisons was performed for more than 2-group comparisons with confidence limits of 95% considered significant.

## 5. Conclusions

This study was focused on the development and evaluation of in vitro 3D RWM models as a potential tool for the rapid development and screening of topically deployable oto-therapeutics. We showed that an RWM outer-layer-only model is sufficient to recapitulate in vitro the drug permeation properties of the native tissues [26], and that these models can be reproducibly used to assess the transmembrane permeation potential of various drugs. Overall, as highlighted already by numerous other research areas [15,16,17,18,19,20,21,22], in vitro 3D models represent a viable and convenient tool for drug discovery advancement, and the RWM model described herein, once validated with human tissue data, could address currently unmet needs in development of topically deliverable oto-therapeutics.

## Data Availability

Data are contained within the article and Supplementary Material.

## Supplementary Materials

The following supporting information can be downloaded at: https://www.mdpi.com/article/10.3390/ph15091105/s1, Table S1: Table for the pertinent physiochemical properties of model pharmaceuticals; Figure S1: Tissue growth time effect on RWM model drug permeation; Figure S2: Tissue freeze/thawing has a significant effect AET TEER.
