# Supplementary material for: Development and Characterization of an In Vitro Round Window Membrane Model for Drug Permeability Evaluations
Source: Pharmaceuticals (Basel). 2022 Sep 5;15(9):1105. doi: 10.3390/ph15091105 (PMC9504332; doi:10.3390/ph15091105)
Supplement: Supplementary file 1 [file pharmaceuticals-15-01105-s001.zip › pharmaceuticals-1865241-supplementary.pdf]

Supplemental Information

Development and Characterization of an In Vitro Round Window Membrane Model for Drug Permeability Evaluations

Ruby Singh[1,2], Bhaskar Birru [1,2], Joachim G.S. Veit [1,2], Elizabeth M. Arrigali [1,2], Monica A. Serban [1,2]

[1] Department of Biomedical and Pharmaceutical Sciences, University of Montana, Missoula, MT 59812, USA

[2] Montana Biotechnology Center (BIOTECH), University of Montana, Missoula, MT 59812, USA

Table S1. Table for the pertinent physiochemical properties of model pharmaceuticals used in the manuscript

Drug	Molecular weight (g/mol)	Chemical Formula	log P
Fluorescein	332.3	$C_{20}H_{12}O_5$	3.88
Dexamethasone Sodium Phosphate	516.405	$C_{22}H_{30}FO_8P$	1.56
Ciprofloxacin HCl	367.8	$C_{17}H_{21}ClFN_3O_4$	-0.86
Gentamicin sulfate	1488.8	$C_{60}H_{125}N_{15}O_{25}S$	-3.1

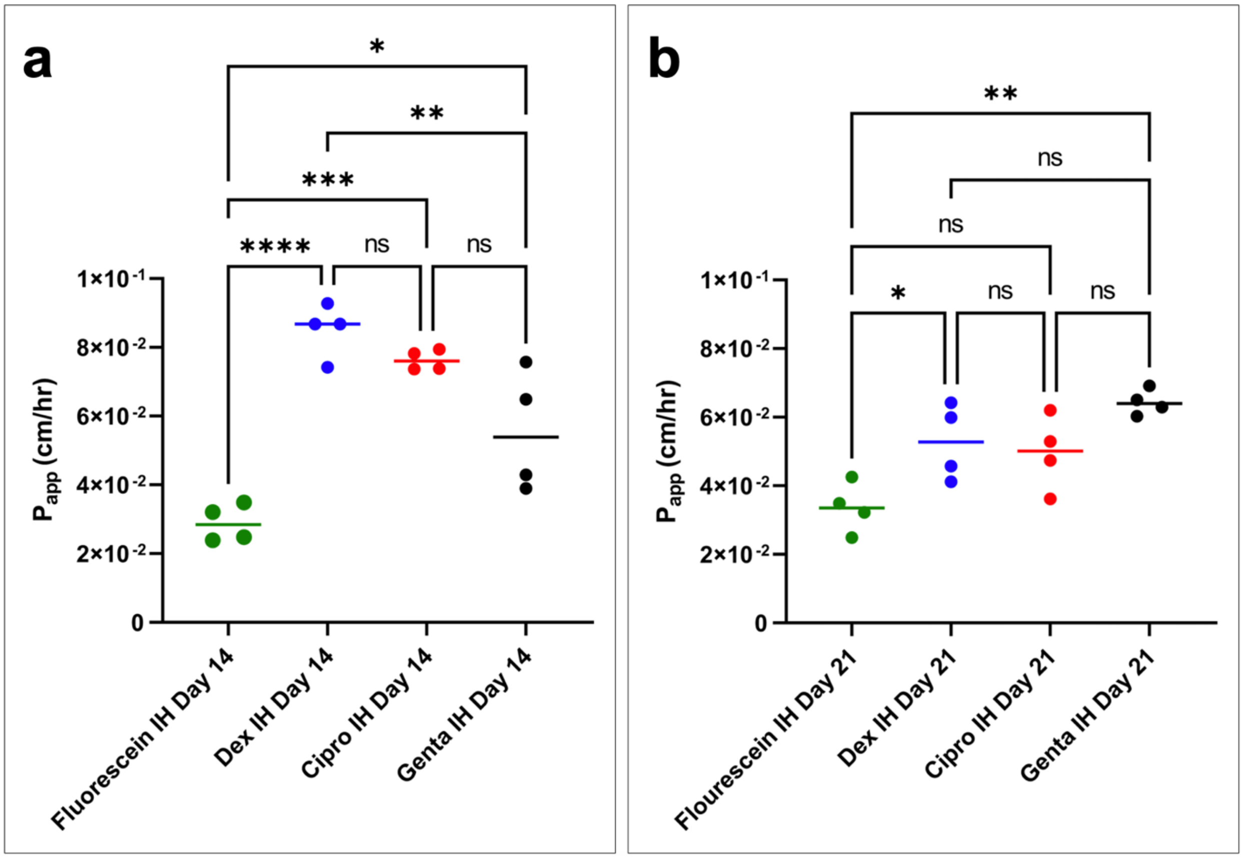

Figure S1. **Tissue growth time effect on RWM model drug permeation.** Drug flux of profile for fluorescein, DSP, ciprofloxacin HCl, and gentamicin sulfate in (**a**) day 14; and (**b**) day 21 AETs. n = 4. One-way ANOVA with Tukey's correction for multiple comparisons; where ***p<0.001, ****p<0.0001, **p<0.001, *p<0.01 as value of significance.

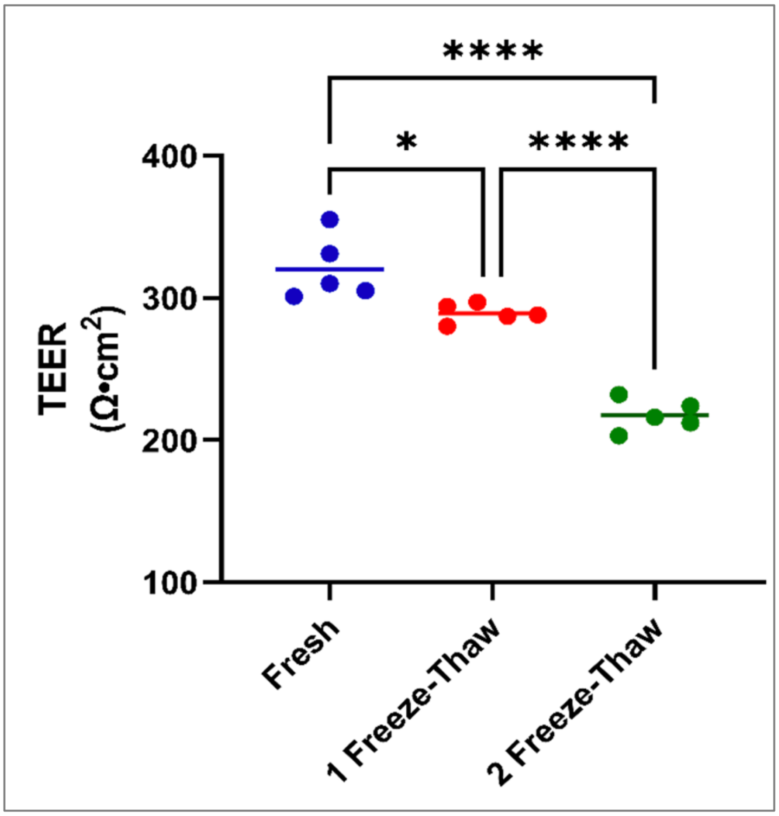

Figure S2. **Tissue freeze/thawing has a significant effect AET TEER.** Effect of freeze/thaw processing cycles on tissue TEER values (n = 5), One-way ANOVA with Tukey’s correction for multiple comparisons, where * ***p<0.0001, *p<0.01 as value of significance.
